# Analysis of the illicit tobacco market in Georgia in response to fiscal and non-fiscal tobacco control measures

**DOI:** 10.1136/tobaccocontrol-2020-056404

**Published:** 2021-06-10

**Authors:** Megan Little, Hana Ross, George Bakhturidze, Iago Kachkachishvili

**Affiliations:** 1 School of Economics, Research Unit on the Economics of Excisable Products, University of Cape Town, Rondebosch, Western Cape, South Africa; 2 Tobacco Control Research, FCTC Implementation and Monitoring Center in Georgia, Tbilisi, Georgia; 3 Sociology and Social Work Department, Ivane Javakhishvili Tbilisi State University, Tbilisi, Georgia

**Keywords:** economics, illegal tobacco products, low/middle-income country, taxation, public policy

## Abstract

**Background:**

Georgian illicit cigarette consumption was 1.5% in 2017. In 2018, a new tobacco control law took effect followed by a substantial cigarette excise tax increase in 2019. Research shows these policies reduce tobacco consumption, but the tobacco industry argues they increase illicit trade. There is limited evidence on this, particularly from developing countries.

**Methods:**

A panel household survey in Georgia obtained data over three waves: 2017 baseline, 2018 after the tobacco control law took effect and 2019 after taxes increased. A sample of 1578 smokers (and quitters in later waves) from five regions reported their tobacco use and were asked to present a cigarette pack in their possession. These were examined for tax stamps and health warnings to establish legality.

**Findings:**

There was no evidence of an increase in illicit cigarette consumption in Tbilisi, Kutaisi, Akhaltsikhe or Gori in any wave. In Zugdidi, near the Russian-occupied Abkhazia, illicit cigarette consumption was increasing even prior to the tax increase, reaching 30.9% by wave 3. A country-wide shift occurred from manufactured cigarettes to roll-your-own tobacco (whose tax remained unchanged) between waves 2 and 3.

**Conclusion:**

No evidence of a country-wide increase in illicit cigarette trade was found after non-fiscal tobacco measures took effect and cigarette taxes increased. Relatively high illicit cigarette consumption in Zugdidi highlights the role of disputed territories and border administration in illicit cigarette supply. Substitution towards roll-your-own tobacco after manufactured cigarette taxes increased demonstrates the importance of equalising taxes on tobacco products to maximise public health benefits.

## Introduction

Georgia has high tobacco use prevalence, estimated at 55% for men and 12% for women in 2016.^1^ To reduce tobacco use and improve public health, the government passed a new tobacco control law in 2017 which came into effect in 2018, and increased cigarette excise tax in 2019.

The new tobacco control law introduced pictorial health warnings, banned smoking in almost all public places and severely limited tobacco advertising.^2^ The 2019 tax reform equalised the specific excise tax rate on filtered and unfiltered cigarettes by increasing the unfiltered tax rate 2.8 times (from 60 tetri to 1.70 Georgian lari (GEL) per 20 cigarettes) and increased the ad valorem tax rate from 10% to 30% on filtered cigarettes.^3^

While the impact of higher tobacco taxes on lowering tobacco use has been widely documented,^4^ there is limited research on the effect of fiscal and non-fiscal policies on illicit tobacco trade. The tobacco industry assertions that tobacco tax increases will only increase illicit tobacco consumption make governments hesitant to increase taxes.^4^

Recent studies showed either no link or a weak link between tax increases and consumption of illicit cigarettes. For example, in Mongolia the share of illicit consumption fell after import and excise taxes rose in 2017 and 2018, respectively.^5^ Despite substantial tax increases in Latin America during the 2000s, there was no statistical evidence of illicit tobacco trade increases in Colombia or Peru.^6^

In the early 2000s, the illicit cigarette market was rampant in Georgia reaching one-third to two-thirds of the total cigarette market.^7^ Since then, the illicit cigarette market share has fallen dramatically to an estimated 1.5% of the total market in 2017 (research used the same wave 1 data used in this paper).^8^ This was the result of improved tax administration and reforms to the country’s revenue and custom services.^4^ The reduction in the illicit cigarette market share coincided with increases in Georgia’s tobacco tax rates.^8^ However, the direct impact of higher excise tax on the illicit tobacco market in Georgia has not been studied yet.

Employing household-level data, this paper examines the effect of two events on the illicit cigarette market in Georgia: the implementation of a new tobacco control law in 2018 focusing on non-fiscal measures, and a substantial tobacco excise tax increase in 2019.

## Methodology

**Figure 1:**
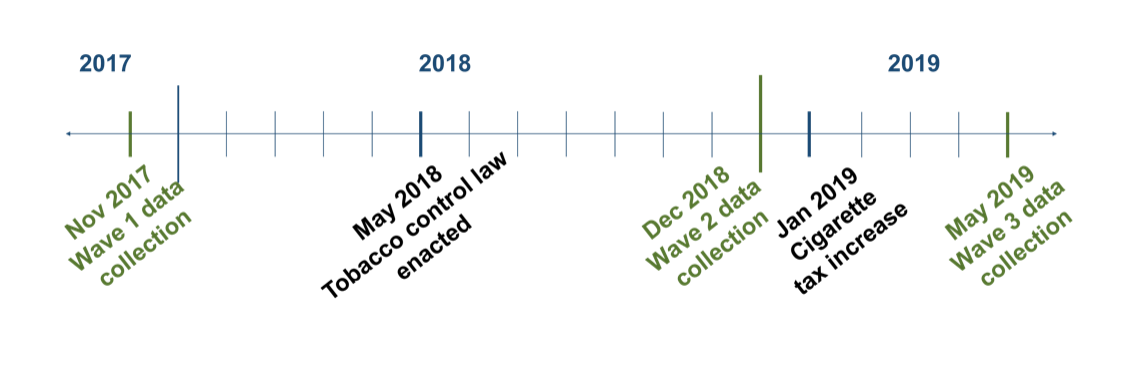
Project timeline.

As shown in figure 1, a panel of smokers (and former smokers in follow-up waves) located in five regions of Georgia (Tbilisi, Kutaisi, Zugdidi, Akhaltsikhe and Gori) was surveyed three times: in November 2017, in December 2018 (after the tobacco control law was enacted in May 2018) and in May 2019 (after the cigarette tax was increased in January 2019). Our choice of the survey method to study illicit trade in Georgia was informed by a systematic review of various methodologies^9^ and the budget available for the study.

**Figure 2:**
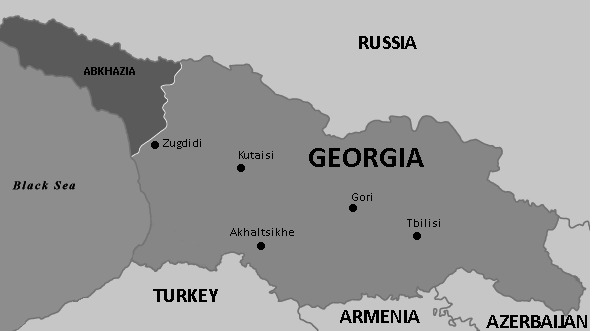
Map of Georgia.

The five regions surveyed represented the geographical diversity of Georgia and/or likely illicit trade hot spots given their proximity to borders (see figure 2). In 2017, the total Georgian population was 3 726 400, with 1 145 500 living in the city of Tbilisi, 142 800 in Kutaisi municipality, 104 200 in Zugdidi municipality, 39 300 in Akhaltsikhe municipality and 123 800 in Gori municipality.^10^ These five regions therefore represented 41.2% of the total Georgian population. In 2017, our survey interviewed 2997 individuals (0.1% of the total Georgian population) with 997 (0.01%) individuals in Tbilisi, 498 (0.4%) in Kutaisi, 500 (0.5%) in Zugdidi, 500 (1.3%) in Akhaltsikhe and 502 (0.4%) in Gori.

Respondents provided basic demographic information and reported the type of tobacco used, frequency, quantity and knowledge of illicit tobacco consumption in their region. In addition, respondents were asked to show a cigarette pack to be examined for brand, price, and the presence of a Georgian tax stamp and health warning. The surveys conducted in all three waves were similar in format, with a few extra questions added in wave 3 to ascertain any behavioural changes resulting from the new law and tax increase.

The sampling began with urban and rural stratification, followed by the selection of primary sampling units (census units in urban strata and villages in rural strata) proportional to population size. Secondary sampling units (households) were then selected using the ‘random step method’—adopted for its cost-effectiveness and because there were households in Georgia without addresses.

If no smokers resided in the selected household, a short general survey was administered to the first adult person answering the door. If the household had a smoker, all smokers were listed and one was randomly selected for the interview. If the selected respondent refused or was not home during any of the three interview attempts, another smoking resident was selected. If no smoking residents were available for interview, the household was recorded as ‘non-responding’ and the interviewer walked past five households on average (the number varied slightly for urban and rural strata) and selected a replacement household.

Table 1 shows that surveyors completed 2997 interviews in wave 1. Of those, 1765 people were successfully re-interviewed in wave 2. These were supplemented with new households (also selected using the ‘random step method’) which brought the total people interviewed in wave 2 to 3040. In wave 3, there were 1578 people interviewed in both waves 1 and 2 (our panel), 240 people interviewed in wave 1 but not in wave 2, 1036 people only interviewed in wave 2, and 335 people added in wave 3 for the first time. As such, there were 3189 people interviewed in wave 3. For quality control, an independent controller cross-checked a randomly selected 10% of completed interviews in each wave and no major errors were identified.

**Table 1:** Response rates by wave

	Wave 1		Wave 2		Wave 3	
Interviewed	2997	68.9%	3040	70.8%	3189	76.7%
No tobacco users reside	957	22.0%	191	4.5%	384	9.2%
Non-respondents	390	8.9%	1061	24.7%	584	14.1%
Total	**4344**		**4292**		**4157**	

Non-respondents included those who refused to participate, were not home or had moved away since a previous wave.

The total attrition in our panel was 52.6% with attrition varying significantly by region. The highest rate was in Tbilisi (62.7%) followed by Kutaisi (52.2%), Akhaltsikhe (47.4%), Gori (31.9%) and Zugdidi (27.4%). Within each region, there was no significant difference in the likelihood of attrition by gender, employment, the probability of showing a pack or the probability of owning an illicit pack. Given the attrition patterns, the analysis was done by region wherever possible.

To establish the rates of illicit cigarette consumption, those respondents who indicated they smoked cigarettes were asked to show the interviewer an available cigarette pack. On examination, the packs lacking either a health warning or tax stamp (required by law^4^) were defined as illicit. Georgia’s tax stamps are secured by a proprietary technology and cannot be removed from a pack without damaging both the packs and the tax stamps.^11^ All statistics extrapolated to be representative of the populations in the five regions are followed by CIs at the 95% level to order to establish statistical significance.

We explored whether illicit cigarette consumption was correlated with changes in both fiscal and non-fiscal measures by comparing illicit cigarette consumption rates at the baseline with the midline (after the non-fiscal changes) and with the endline (after the tax increase). Even though the broader economic environment in Georgia was fairly stable during this period with the real gross domestic product growth between 4.8% and 5%,^12^ and the average annual inflation between 3% and 5%,^13^ we cannot ascertain causation.

In addition to the household survey, qualitative data were gathered through three focus group discussions (FGDs) with 8–10 participants in each group in Tbilisi, Gori and Zugdidi in December 2018 to obtain additional context of both licit and illicit cigarette consumption. The participants were of both genders, different ages and social status, and were mainly smokers or people from smokers’ families. The purpose of the focus groups was to understand why a relatively large percentage of respondents refused to show their cigarette packs to a surveyor. This informed a small change in the questionnaire in waves 2 and 3, where respondents were asked about their cigarettes’ tar content immediately before being asked to show a pack, to encourage people to show packs by shifting the focus from illicit cigarettes (and a possible unease about smoking them).

## Results

We present results for the respondents who responded to all three surveys (the panel), unless otherwise stated. The demographic characteristics of all panel respondents in wave 1 (extrapolated to the population) are presented in table 2. There was no evidence that demographics were statistically significantly correlated with attrition.

**Table 2:** Percentage of people, by demographic characteristic in wave 1

	Wave 1 (CI)
Gender: male (%)	89.7 (88.1 to 91.1)
Age bracket: 18–29 years (%)	19.7 (17.8 to 21.7)
Age bracket: 30–49 years (%)	46.2 (43.8 to 48.7)
Age bracket: 50 years and older (%)	34.2 (31.9 to 36.5)
Education: less than secondary school (%)	6.6 (5.5 to 7.9)
Education: secondary school complete (%)	43.3 (40.9 to 45.8)
Education: vocational education complete (%)	12.1 (10.5 to 13.8)
Education: tertiary education incomplete (%)	3.9 (3.1 to 5.0)
Education: tertiary education complete (%)	34.1 (31.9 to 36.5)
Mean household size (number of people)	3.6 (3.5 to 3.6)
Employment status: employed in private or public sector (%)	28.4 (26.2 to 30.7)
Employment status: self-employed (%)	31.9 (29.6 to 34.3)
Employment status: unemployed (%)	29.7 (27.5 to 32.0)
Employment status: not in labour force (%)	10.0 (8.6 to 11.6)

Between waves 1 and 2 (360 days apart), 9.1%, CI (7.9% to 10.5%) of wave 1 smokers had given up smoking. By wave 3 (500 days after wave 1), nearly two-thirds of these people remained non-smokers: 5.6%, CI (4.7% to 6.8%) of wave 1 smokers were still non-smokers in wave 3, while 3.5%, CI (2.7% to 4.4%) of wave 1 smokers quit in wave 2 but re-initiated smoking by wave 3. Among people who smoked in both wave 1 and wave 2, 5.2%, CI (4.3% to 6.4%) had given up smoking by wave 3 (140 days after wave 2). There was no evidence that the reported quit rates were affected by attrition bias.

Table 3 shows that the main reasons for quitting reported in wave 2 (after the new tobacco control law came into effect) were health (48.5%) and affordability (44.3%), while 7.2% cited other reasons (predominantly that they did not know the reason they quit). Wave 2 smokers who gave up smoking by wave 3 (after the tax increase) cited affordability as the predominant cause for quitting. In total, nearly 75% of those who quit in wave 3 cited either only cost or both cost and health as their reason for quitting, while 42% cited only health or both cost and health as their reason. Nearly 9% of wave 3 quitters cited other reasons such as inconvenience due to the public smoking ban or disliking the negative health images on packs.

**Table 3:** Percentage of people, by reason for quitting smoking since previous wave

	Reported in wave 2	Reported in wave 3
Health	48.5%, CI (42.2% to 54.9%)	16.3, CI (11.1% to 23.3%)
Cost	44.3%, CI (38.1% to 50.7%)	49.0%, CI (40.9% to 57.1%)
Both health and cost	N/A	25.8%, CI (19.4% to 33.6%)
Other	7.2%, CI (4.5% to 11.3%)	8.8%, CI (5.2% to 14.7%)

The survey was administered slightly differently in wave 2 and wave 3: respondents could select only one reason in wave 2 while in wave 3 they could select more than one reason.

N/A, not available.

Roll-your-own (RYO) tobacco increased in popularity, particularly after the cigarette tax increase between waves 2 and 3 (see table 4). While nearly all tobacco users in the five regions consumed only manufactured cigarettes in wave 1 and wave 2, by wave 3 this share had dropped and the share of people consuming RYO tobacco increased. In all three waves, the majority of manufactured cigarettes consumed were imported: 81.4%, CI (79.4% to 83.3%) of manufactured cigarettes were imported in wave 1; 80.3%, CI (78.2% to 82.2%) in wave 2 and 91.7%, CI (90.0% to 93.2%) in wave 3.

**Table 4:** Percentage of people, by tobacco type consumed each wave

	Wave 1	Wave 2	Wave 3
Manufactured cigarettes	97.3%, CI (96.5% to 98.0%)	96.3%, CI (95.3% to 97.2%)	72.2%, CI (70.0% to 74.4%)
Manufactured cigarettes and RYO	1.0%, CI (0.6% to 1.7%)	0.5%, CI (0.3% to 1.0%)	4.3%, CI (3.4% to 5.5%)
RYO only	1.0%, CI (0.6% to 1.7%)	2.8%, CI (2.1% to 3.7%)	23.3%, CI (21.3% to 25.5%)
Only other tobacco	0.7%, CI (0.4% to 1.3%)	0.4%, CI (0.2% to 0.9%)	0.1%, CI (0% to 0.4%)

RYO, roll-your-own.

Wave 3 smokers were asked how the new tobacco control law and tax increase impacted their behaviour. In response to the new law, about 49.0%, CI (47.2% to 50.1%) of wave 3 smokers reported no change in behaviour; 17.6%, CI (16.3% to 19.0%) smoked at home instead of in public; 16.7%, CI (15.4% to 18.1%) switched to another type of tobacco product; 1.5.%, CI (1.1% to 2.0%) continued to smoke in public places illegally; and 1.4%, CI (1.1% to 1.9%) consumed less tobacco. Only 3.5%, CI (2.9% to 4.2%) of smokers were unaware of the new law.

Regarding the tax increase, 5.2%, CI (4.5% to 6.1%) of wave 3 smokers were unaware the tax had increased; 43.3%, CI (41.7% to 45.1%) kept their behaviour unchanged; 21.0%, CI (19.5% to 22.3%) consumed less tobacco; 14.0%, CI (12.8% to 15.2%) chose to shop in a new location or buy a new brand; and 11.7%, CI (10.6% to 12.9%) changed to RYO.

In each wave, respondents who smoked manufactured cigarettes were asked to show surveyors their cigarette pack (see table 5). In wave 1, 74.2% showed a pack, which rose to 82.7% in wave 2. However, this percentage fell to 67.2% in wave 3. The highest level of cooperation was in Tbilisi, while the lowest was in Zugdidi, a city in the western part of the country close to Abkhazia (occupied by Russia). Overall, women and employed respondents were more willing to show their packs than men and unemployed respondents. There was no substantial difference in proportion of respondents that showed packs by age.

**Table 5:** Percentage of respondents that showed packs, by wave

	Wave 1	Wave 2	Wave 3
Total	74.2%	82.7%	67.2%
Tbilisi	85.8%	88.3%	83.7%
Kutaisi	72.7%	77.6%	64.2%
Zugdidi	63.2%	74.6%	48.1%
Akhaltsikhe	78.7%	90.1%	72.0%
Gori	70.6%	83.3%	71.6%
Male	72.5%	81.8%	64.5%
Female	90.0%	90.7%	84.3%
Employed or self-employed	76.6%	85.0%	70.0%
Unemployed	68.3%	78.6%	60.0%

The FGDs suggested possible reasons for the hesitation to show a pack which included embarrassment to show a cheap brand if respondents had reported smoking a more expensive brand, or that respondents were attempting to hide their smoking habit thus had no pack at home (particularly among women and youth). Some participants in Tbilisi speculated that the reason was to avoid showing an illicit pack, but they also noted that they had not seen illicit cigarettes in Tbilisi for a long time. The FGD in Zugdidi suggested that respondents may conceal an illicit pack to protect the vendor from which they bought it, although they were unlikely to worry about any personal consequences for having one illicit pack.

All available packs were examined and classified as illicit if they lacked either the Georgian tax stamp or the Georgian health warning. Robustness checks were performed and the illicit consumption results did not differ statistically significantly between all packs versus packs just from the panel, or when we defined packs with Georgian health warnings but missing or unclear tax stamps as licit. This was true for the overall sample and for all regions.

In waves 2 and 3, there were 9 and 27 packs, respectively, that had Georgian tax stamps and English health warnings. This is only legal for Duty Free packs, however none of these packs were found in Tbilisi where Duty Free shops are located. In both waves, over 75% of these packs were recorded by the same interviewer and as such we assumed this was an error and these packs had their illicit status marked as missing. A sensitivity analysis was conducted, and no statistically significant difference was found in the percentage of illicit packs by region when these packs were defined as licit.

**Figure 3:**
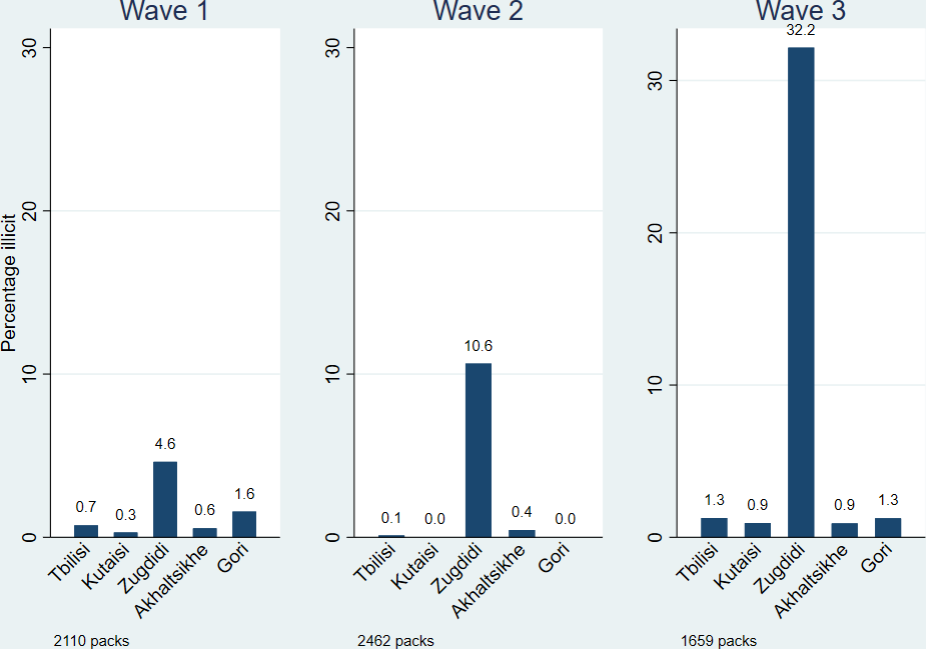
Percentage of illicit cigarette packs, by region.

Figure 3 shows that there was no statistically significant rise in the proportion of illicit packs in Tbilisi, Kutaisi, Akhaltsikhe or Gori between waves 1 and 3. In Zugdidi, the percentage of smokers with illicit packs rose from 4.6%, CI (2.7% to 7.8%) in wave 1 to 10.6%, CI (7.8% to 14.3%) in wave 2, and further to 32.2%, CI (26.0% to 39.0%) in wave 3. Within Zugdidi, there was no observed statistically significant difference in the ownership of illicit packs by employment status or age, and the gender difference could not be determined due to the limited number of observations.

**Figure 4:**
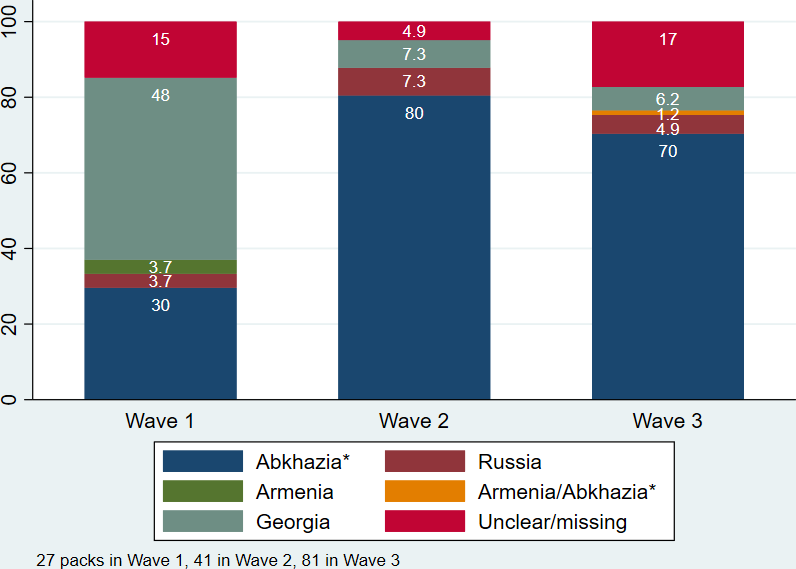
Percentage of illicit packs by country of origin, by wave.

All illicit packs were further examined first for foreign tax stamps and then for foreign language health warnings. This yielded a probable country (or occupied territory) of origin for most illicit packs. There were 27 illicit packs in wave 1, 41 in wave 2 and 81 in wave 3. The proportion of illicit packs originating from Abkhazia was 29.6%, CI (14.8% to 50.4%) in wave 1 (8 packs); 80.5%, CI (64.8% to 90.2%) in wave 2 (33 packs); and 70.4%, CI (59.3% to 79.5%) in wave 3 (57 packs) (figure 4). While the increase from wave 1 to 2 was statistically significant, the decrease from wave 2 to 3 was not. The percentage of packs that had Georgian health warnings but no tax stamp fell from 48.2%, CI (29.3% to 67.5%) in wave 1 (13 packs), to 7.3%, CI (2.3% to 21.2%) in wave 2 (3 packs), and 6.2%, CI (2.5% to 14.2%) in wave 3 (5 packs).

Among licit cigarette packs, the most popular brands were Winston (imported) and Pirveli (domestically produced). On average across the waves, Winston represented 13.0%, CI (12.2% to 13.9%) of the market and Pirveli represented 14.9%, CI (14.0% to 15.8%), while no other brands represented more than 10% of the market. Table 6 provides information on the illicit cigarette brands. Most illicit packs were international brands but there were some Georgian brands classified as illicit due to a missing tax stamp. The Manchester brand, clearly dominates, representing 33.3% of all illicit packs in wave 1, 83.0% in wave 2 and 66.3% in wave 3. In all three waves, the illicit Manchester packs were found only in Zugdidi and the majority had an Abkhazian tax stamp.

**Table 6:** Illicit cigarette brands by location of the tax stamp’s origin, by wave

	Wave 1	Wave 2	Wave 3	Total
Armada	1 (unclear/missing)		7 (6 unclear/missing, 1 Georgia)	8
Astra	1 (Georgia)		2 (unclear/missing)	3
Camel Blue			1 (Georgia)	1
Chibukh	1 (Georgia)	1 (Georgia)		2
Continent		2 (1 Abkhazia territory, 1 Russia)	1 (Abkhazia territory)	3
Get	2 (Abkhazia territory)			2
GM		1 (unclear/missing)		1
Imperator			1 (unclear/missing)	1
Kent			1 (Georgia)	1
L&M	4 (Georgia)			4
Magna	1 (Georgia)		1 (Georgia)	2
Manchester	9 (6 Abkhazia territory, 1 Russia, 2 unclear/missing)	34 (31 Abkhazia territory, 2 Russia, 1 unclear/missing)	53 (49 Abkhazia territory, 4 Russia)	96
Marlboro	1 (unclear/missing)		2 (unclear/missing)	3
MZE (White)		1 (Georgia)		1
M1		1 (Abkhazia territory)	3 (Abkhazia territory)	4
Phillip Morris	1 (Georgia)		1 (unclear/missing)	2
Pirveli	3 (Georgia)	1 (Georgia)		4
Prima	1 (Georgia)			1
Samefo			1 (unclear/missing)	1
VIP	1 (Armenia)			1
Wilson Blue			1 (unclear/missing)	1
Winston			1 (Georgia)	1
X1 Silver Slims	1 (Georgia)			1
Unknown Armenian/Abkhazian brand			5 (4 Abkhazia territory, 1 Armenia)	5
	**27**	**41**	**81**	**149**

**Figure 5:**
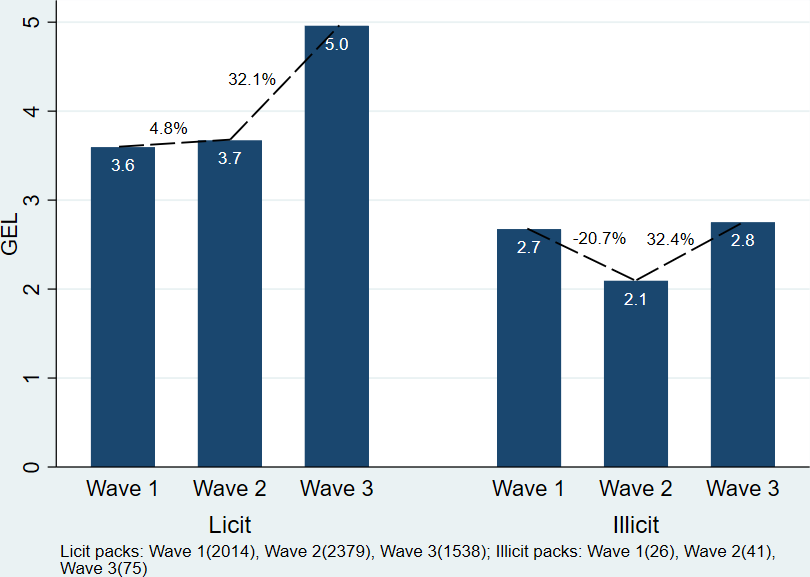
Average price for illicit and licit packs (20 cigarettes) per wave, and price changes. GEL, Georgian lari.

The analysis of price per pack (self-reported by the smoker) in figure 5 shows that the average price of the licit packs was GEL 3.6, CI (3.5 to 3.6) in wave 1; GEL 3.7, CI (3.6 to 3.7) in wave 2 and GEL 5.0, CI (4.9 to 5.0) in wave 3. In comparison, the average price of the illicit packs was GEL 2.7, CI (2.3 to 3.0) in wave 1; GEL 2.1, CI (2.0 to 2.2) in wave 2 and GEL 2.8, CI (2.4 to 3.1) in wave 3. In wave 2, the average price of licit packs rose by 4.8%, CI (0.4% to 8.1%), while the average price of illicit packs fell by 20.7%, CI (–41.2% to –8.0%). After the tax increase (between waves 2 and 3), the price of licit and illicit packs rose by 32.1%, CI (10.0% to 58.8%) and 32.4%, CI (0.7% to 63.4%), respectively. The average annual national consumer price indices for imported manufactured cigarettes were 8.6% from 2017 to 2018, and 28.9% from 2018 to 2019.^14^

Table 6 indicates the illicit brands observed in each wave varied. To test whether the average price changes of illicit packs were driven by smokers switching brands or price changes within each brand, we examined the prices of the most popular illicit brand, Manchester. In addition, we analysed price changes for the two most popular licit brands, Winston and Pirveli. On average, illicit Manchester packs were GEL 2.3, CI (2.0 to 2.7) in wave 1; GEL 2.1, CI (2.0 to 2.1) in wave 2 and GEL 2.6, CI (2.5 to 2.7) in wave 3. Therefore, the prices of illicit Manchester packs on average fell by 10.1%, CI (−7.3% to –15.3%) from wave 1 to 2, and rose by 24.6%, CI (16.5% to 31.0%) from wave 2 to 3. For licit packs, the price of a pack of Winston rose by 10.3%, CI (5.4% to 13.6%) from wave 1 to 2, and by 22.7%, CI (15.1% to 29.1%) from wave 2 to 3. Packs of Pirveli rose by 8.0%, CI (2.6% to 12.1%) from wave 1 to 2, and by 34.2%, CI (16.0% to 54.4%) from wave 2 to 3. Price outliers at the 1st and 99th percentile for licit and illicit packs in each wave were excluded for this analysis, to avoid any bias from data capture error.

## Discussion

Between November 2017 and May 2019, the long-term smoking cessation rate was at least 5.6%. This result needs to be interpreted with caution since our sample was not representative of the Georgian population. The rate of cessation was most likely dampened by a shift towards cheaper RYO, particularly after the tax increase. In November 2017, only 1.0% of smokers consumed only RYO. By May 2019, this share increased to 23.3% with smokers reporting affordability as the main reason for their switch in wave 3. The government addressed this substitution by increasing excise tax on RYO products from GEL 35 to GEL 60 per kg in November 2019.

Illicit cigarette consumption was low and did not change significantly in Tbilisi, Kutaisi, Akhaltsikhe and Gori between waves 1 and 3. In Zugdidi, consumption of illegal cigarettes increased substantially from wave 1 to 3, but this trend already began prior to the tax increase. Zugdidi is located near Abkhazia, the Russian-occupied region, and the Manchester brand was the most common illicit brand dominating all three waves and it was found only in the Zugdidi region. There are still close ties between the regions of Zugdidi and Abkhazia—people are crossing the border back and forth regularly and the border administration is weak. Based on the information received during our FGDs, people buy Manchester cigarettes in Abkhazia, then either bribe the border guards or walk through unchecked. These cigarettes are then sold in markets in Georgia. In all three waves, less than 10% of illicit packs originated from the neighbouring countries of Russia, Armenia, Turkey and Azerbaijan.

Gori is located near Georgia’s second occupied region, South Ossetia, however the situation was different here and we found almost no illicit cigarettes in Gori. Similarly, despite Akhaltsikhe being located near the border with Armenia where cigarettes are cheaper than Georgia,^4^ there were almost no illicit cigarettes in Akhaltsikhe. This points to strong levels of enforcement in these vulnerable areas of Georgia, relative to the Zugdidi region. On average, the prices of illegal cigarettes responded to the tax increase by going up by the same percentage as the legal cigarettes between wave 1 and wave 3, even though they were still about 50% cheaper in wave 3 compared with their legal counterparts. The most prevalent illicit brand, Manchester, was cheaper than other illicit brands, and its price changes were more muted compared with other illicit brands. Thus, there is some evidence that smokers of illicit cigarettes may have switched to the Manchester brand for affordability.

Our study has several limitations. First, our sample is not representative of the Georgian population. However, we covered five regions of Georgia to capture any regional differences. Second, not everybody agreed to show a cigarette pack to the surveyor. If the reason was the possession of an illicit pack, then the size of the illicit cigarette market will be biased downwards. This bias may be significant given the low levels of cooperation in Zugdidi, the region with the highest level of illicit consumption. However, respondents were informed that the survey was anonymous to reduce fear of incrimination, and the information from the focus group suggests that the possession of an illicit pack was just one reason for not showing a pack. Further research using littered pack collection in the region of Zugdidi is recommended, because this methodology is free of bias related to the willingness to show a pack. Third, even though we used the presence of a Georgian tax stamp as one of the signs of a legal pack, the authenticity of tax stamps was not tested due to limited budget. Information from the Georgia Customs office received in September 2019 indicates a rare occurrence of counterfeit tax stamps in Georgia. Finally, our study did not assess the legality of RYO, thus we cannot comment on the share of illicit tobacco consumed, only on illicit cigarette consumption. We recommend further research on illicit RYO.

## Conclusion

The adoption of fiscal and non-fiscal tobacco control policies in Georgia had no impact on illicit cigarette consumption due to the presence of effective tax administration and enforcement. The illicit cigarette market in Zugdidi that was present already before the adoption of the new policies highlights the importance of war zones and disputed territories in the supply of illicit cigarettes. Georgia should consider tightening controls with Abkhazia and ratifying the Framework Convention on Tobacco Control Protocol to Eliminate Illicit Trade in Tobacco Products as this would strengthen its efforts to control the illicit cigarette market. It will be important to monitor if higher excise taxes on RYO addressed the recent movement from manufactured cigarettes to RYO cigarettes, and further reduced the smoking prevalence. We recommend further exploration of the impact of the tax increase and other policy changes on illicit tobacco market in Georgia using multiple methods and nationally representative data.

**What this paper adds**

**What is already known on this subject**

- In Georgia and elsewhere, the tobacco industry argues that increasing cigarette taxes leads to a rise in illicit cigarette consumption. A study published in this journal in 2020 showed that the illicit cigarette penetration in Georgia was low despite recent tobacco tax increases. However, it could not establish a relationship between a change in tax and a change in the cigarette illicit trade.

**What this paper adds**

- This study consistently measures the size of illicit cigarette market in five regions of Georgia from 2017 to 2019, a period when both new non-fiscal policies and higher taxes were implemented. The results show no country-wide increase in illicit tobacco consumption but point to a regional problem of an illicit cigarette market that persists over time and is related to the disputed border area occupied by Russia. We conclude that illicit cigarette trade in Georgia is not related to tax changes or changes to other tobacco control policies, but to border security.

## Data Availability

We will be making the data collected during our three waves of data collection in Georgia publicly available through the University of Cape Town’s DataFirst as our data publishing repository and the data will be deidentified with no personally identifiable information of the participants made publicly available.
